# Infiltration of LPAR5^+^ macrophages in osteosarcoma tumor microenvironment predicts better outcomes

**DOI:** 10.3389/fimmu.2022.909932

**Published:** 2022-12-15

**Authors:** Yi He, Haiting Zhou, Xiaojian Huang, Yunkun Qu, Yingguang Wang, Wenbin Pei, Rui Zhang, Sheng Chen, Hongbo You

**Affiliations:** ^1^ Department of Orthopedics, Tongji Hospital, Tongji Medical College, Huazhong University of Science and Technology, Wuhan, Hubei, China; ^2^ Department of Oncology, Tongji Hospital, Tongji Medical College, Huazhong University of Science and Technology, Wuhan, Hubei, China

**Keywords:** tumor microenvironment, macrophage, estimate, CIBERSORT, osteosarcoma, LPAR5

## Abstract

**Introduction:**

Tumor microenvironment (TME) has been shown to be extensively involved in tumor development. However, the dynamic change of TME components and their effects are still unclear. Here, we attempted to identify TME-related genes that could help predict survival and may be potential therapeutic targets.

**Methods:**

Data was collected from UCSC Xena and GEO database. ESTIMATE and CIBERSORT algorithms were applied to estimate the components and the proportions of TIICs in TME. We analyzed the gene expression differences of immune components and stromal components, respectively, and finally got the overlapped DEGs. Through protein-protein interaction (PPI) network and univariate Cox regression analysis based on shared DEGs, we screened out and validated the TME-related genes. Focusing on this gene, we analyzed the expression and prognostic value of this gene, and investigated its relationship with immune cells by correlation analysis, single cell analysis, immunohistochemistry and immunofluorescence analysis.

**Results:**

Through a series analysis, we found that the proportion of immune and stromal components was an important prognostic factor, and screened out a key gene, LPAR5, which was highly correlated with prognosis and metastasis. And the expression of LPAR5 was positively correlated with immune cells, especially macrophages, indicating LPAR5^+^ macrophages played an important role in tumor microenvironment of osteosarcoma. Meanwhile, the genes in LPAR5 high expression group were enriched in immune-related activities and pathways, and differentially expressed genes between LPAR5^+^ macrophages and LPAR5^-^ macrophages were enriched in the biological processes associated with phagocytosis and antigen presentation. What’ more, we found that LPAR5 was mainly expressed in TME, and high LPAR5 expression predicting a better prognosis.

**Conclusion:**

We identified a TME-related gene, LPAR5, which is a promising indicator for TME remodeling in osteosarcoma. Particularly, LPAR5^+^ macrophages might have great potential to be a prognostic factor and therapeutic target for osteosarcoma.

## 1 Introduction

Osteosarcoma is one of the most common bone tumors, accounting for 20–40% of all bone tumors, and it is most often seen in children and adolescents (1, 2). It occurs mainly in the epiphysis of distal femur, proximal tibia and proximal humerus (3–5). The incidence of osteosarcoma has a bimodal distribution, with the first peak appearing in the adolescent (10-19 years old) and the second peak appearing in the elderly (60 years old). The incidence of osteosarcoma has not changed significantly in recent years (6). In the general population, the incidence of osteosarcoma is 2-3 per million people each year, but it is higher in adolescents, with a maximum incidence of 8-11 per million people each year in adolescents aged 15-19 years (3, 7). Males has higher prevalence rate than that of females (8, 9). Osteosarcomas are prone to local invasion and early metastasis, mostly to the lung. It is reported that 20% of patients are found to have pulmonary metastases at the time of initial diagnosis (10), and tumor recurrence is reported in 30 to 50 percent of patients after treatment (11). Medical advances have significantly improved prognosis for localized osteosarcoma patients, with the average 5-year overall survival rate of 80% (7, 12). But unfortunately, patients with metastasis have a significantly worse prognosis (13, 14). Meanwhile, the lack of specific diagnostic markers makes early screening for osteosarcoma still difficult. Distant metastasis and drug resistance also worse the prognosis of patients (10, 15–17). It is urgent to figure out the underlying pathogenesis of osteosarcoma, and to discover potential targets for earlier diagnosis and potential drugs for better treatment.

Tumor microenvironment (TME) refers to the cellular environment in which tumors or cancer stem cells are located. Besides, the microenvironment also includes components such as adipocytes, fibroblasts, tumor vasculature, tumor-infiltrating immune cells (TIICs) (18, 19). TME has been shown to be extensively involved in tumor development by interacting with surrounding cells through the circulatory and lymphatic systems. Osteosarcoma is no exception (20). The components of TME are mainly resident stromal cells and recruited TIICs. TIICs are the major non-tumor component of the osteosarcoma tumor microenvironment (21). Recent studies suggested that the recruitment, activation, and reprogramming of TIICs were associated with interactions between cancer cells and TME (22). And the change of components of the TME could affect tumor development and progression (23). Therefore, TME components are helpful for evaluations of prognosis and therapeutic effects (24, 25). Furthermore, the intervention and alteration of TME components are potential immunotherapy approaches.

ESTIMATE is an algorithm used to calculate the fraction of immune components and stromal components in tumor samples (26). A series of studies used this algorithm to evaluate the prognostic value of immune components and stromal components in breast cancer, prostate cancer, and melanoma recently (27–29). CIBERSORT is also an algorithm used to assess specific levels of TIICs in tumor samples (30), which has been applied to identify correlations between TIICs proportion and prognosis in colorectal cancer, nasopharyngeal carcinoma and bladder cancer (31–33).

**Figure 1:**
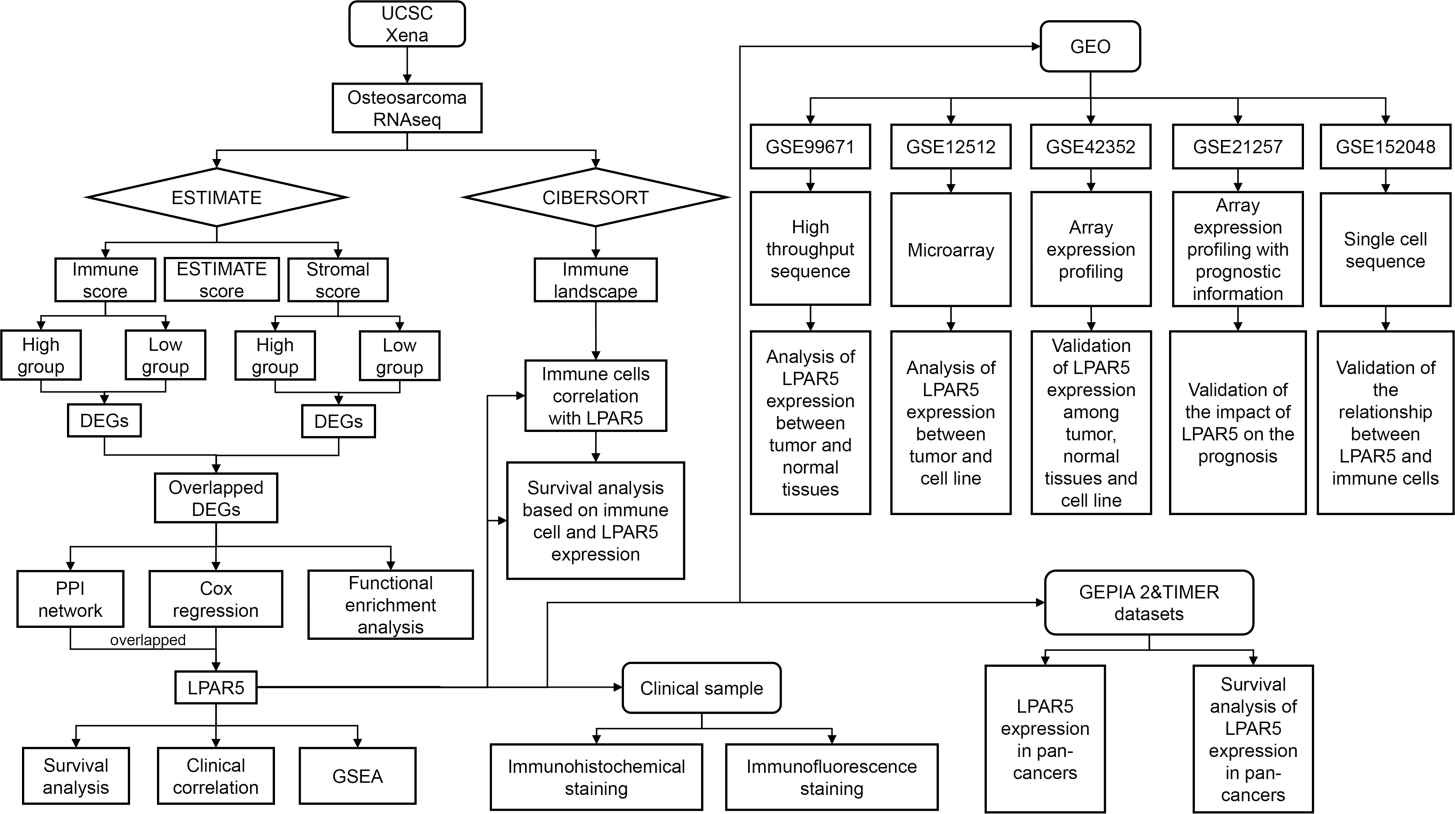
Analysis flow chart of our study.

In our study, we collected the expression profile and corresponding clinical information of osteosarcoma from UCSC Xena database. ESTIMATE and CIBERSORT algorithms were applied to estimate the amount of immune and stromal components and the proportions of TIICs in all samples. We analyzed the gene expression differences of immune components and stromal components, respectively, and finally got the shared DEGs by intersection. Through protein-protein interaction (PPI) network and univariate Cox regression analysis based on shared DEGs, we screened out and validated a TME-related core gene, LPAR5, which may be a promising indicator for TME remodeling with a prognostic value in osteosarcoma. The flowchart of our study is shown in **Figure 1**.

## 2 Methods

### 2.1 Sample datasets

The RNAseq (level 3) data (FPKM) and corresponding clinical data of osteosarcoma were retrieved from the UCSC Xena database (http://xena.ucsc.edu/). Samples with incomplete clinical information and overall survival time lower than 30 days were removed. Eighty-five samples were incorporated into this study. The data is converted to a TPM format. Five GSE datasets, GSE99671 (34), GSE12512, GSE42352 (35) and GSE152048 (36), GSE21257 (37) were collected from the GEO database (https://www.ncbi.nlm.nih.gov/geo/). All the gene expression must greater than 1 in at least two samples, otherwise the gene will be excluded. All data was available to the public, so there was no further approval needed from the Ethics Committee.

### 2.2 ESTIMATE analysis

ESTIMATE analysis (26) was applied to estimate the component of TME by calculating immune score, stromal score and ESTIMATE score for each sample. All samples were divided into the high score and the low score group, depending on the comparison to the median value of the immune score, stromal score and ESTIMATE score. Furthermore, the possible correlation of immune score, stromal score and ESTIMATE score with overall survival were identified by “survival” package and “survminer” package.

### 2.3 Identification of differentially expressed genes

To further identify the DEGs between high and low score group, differential analysis was performed with the “limma” package (38). The cut-off value was set as |log foldchange| >1 and P-value <0.05. Then DEGs obtained from the two score-group were intersected to obtain shared DEGs.

### 2.4 Functional enrichment analysis

Gene Ontology (GO) and Kyoto Encyclopedia of Genes and Genomes (KEGG) were performed to analyze pathways associated with DEGs by using “org.Hs.eg.db”, “clusterProfiler” (39), “enrichplot”, and “ggplot2” packages in R software.

### 2.5 Protein-protein interaction network and cox regression analysis

To further explore these genes’ possible mechanisms and find the core nodes, a PPI network was constructed to further explore possible relationships among these genes by STRING database and then visualized by Cytoscape (version 3.8.0). Confidence of the interactive relationship of nodes in the network was set as 0.90. And top 30 nodes were selected for subsequent analysis. Univariate Cox regression analysis was then performed to screen out the prognostic genes by using the “survival” package. The P-value was set as 0.01. At last, key genes were obtained by intersecting the genes in PPI network and the prognostic genes.

### 2.6 Survival analysis and clinical correlation analysis

According to the median value of LPAR5 expression, patients were divided into high expression group and low expression group. Then survival analysis was performed to investigate the correlation between LPAR5 and survival. Clinical correlation analysis was performed to explore the relationship between LPAR5 and clinical characteristics, such as age, gender and metastasis status, by using Wilcox tests. P<0.05 was considered as statistical significance.

### 2.7 Gene set enrichment analysis

GSEA was carried out to explore pathways that were differentially enriched between high and low LPAR5 expression group by using the GSEA software (version 4.1.0). And the final results were integrated and visualized by “ggplot2” package. P< 0.05 was considered statistical significance. The top 10 immune-related function and pathways with q-value<0.01 were displayed.

### 2.8 CIBERSORT analysis

To further investigate the correlation between the expression level of LPAR5 and immune components in tumor microenvironment, immune-infiltration analysis was performed. The proportion of TIICs was assessed by applying the CIBERSORT deconvolution algorithm (30) in all samples. Then the correlation between LPAR5 and immune cells was investigated by using spearman correlation analysis. P<0.05 was considered as statistically significant.

### 2.9 Survival analysis of LPAR5-expressing immune cells

To further investigate the impact of LPAR5-expressing immune cells on patient prognosis, patients were divided into four groups as follows: 1) High LPAR5 + High immune cell; 2) High LPAR5 + Low immune cell; 3) Low LPAR5 + Low immune cell; 4) Low LPAR5 + High immune cell. Subsequent survival analysis was performed based on these four groups. To test significance, we used t-test for each two group.

### 2.10 Analysis and validation in GEO datasets

GSE99671 dataset was a high throughput sequencing profiling, which contained 18 tumor samples and 18 paired non-tumor samples. The dataset was used to explore the expression difference of LPAR5 between tumor tissues and normal tissues. GSE12512 dataset was a microarray that contained 27 osteosarcoma tumors, 12 osteosarcoma cell lines and 8 leiomyosarcoma tumors. The dataset was used to explore the expression difference of LPAR5 between tumor tissues and cell line. GSE42352 was an array expression profiling composed of 12 mesenchymal stem cells, 84 pre-treatment high-grade osteosarcoma diagnostic biopsies and 19 high-grade osteosarcoma cell lines. The dataset was used to validate the results we found above. Wilcox test was used to test the significance. GSE21257 dataset was a genome-wide gene expression profile comprising pre-chemotherapy biopsies from 34 osteosarcoma patients with metastases within 5 years and 19 osteosarcoma patients without metastases within 5 years. The dataset was used to validate the impact of LPAR5 on the prognosis of patients with osteosarcoma. We analyzed the impact of LPAR5 on the prognosis of patients with osteosarcoma according to the conception of metastasis-free survival as defined by the authors of the dataset (37). The metastasis-free survival was observed on whether the patient had metastasis, and the time it occurred. In contrast, the observation point for conventional survival analysis was whether the patient occurred to die and the time to death. Briefly, we divided the patients into high or low expression groups based on their LPAR5 expression and observed whether there was a difference in metastasis-free survival between the two groups. A single cell RNA-seq transcriptome datasets (GSE152048) (36), which contained seven primary, two recurrent and two lung metastasis osteosarcoma sample, were used for further analysis. The “Seurat” package (version 4.1.0) was used to perform the quality control and main analysis (40). The data was integrated by using the “Merge” function and the batch effect was eliminated by using “Harmony” package (41). The cell markers used to identify cell cluster were collected in study of Zhou et al. (36) and CellMarker dataset (42) (http://bio-bigdata.hrbmu.edu.cn/CellMarker/index.jsp). Macrophages were acquired by using the “Subset” function of the “Seurat” package. Then, macrophages were clustered into LPAR5^+^ macrophages group and LPAR5^-^ macrophages groups based on whether LPAR5 was expressed or not. Possible mechanisms were explored by GSEA analysis.

### 2.11 Immunohistochemical and immunofluorescence staining

To verify the difference in the expression of LPAR5 between tumor tissues and normal tissues and the relationship between LPAR5 and immune cells, immunohistochemical and immunofluorescence staining were performed according to the manufacturers’ protocols. Tumor tissue slides and paraneoplastic tissue slides of osteosarcoma were obtained from the Department of Pathology, Tongji Hospital, Tongji Medical College, Huazhong University of Science and Technology. Anti-CD8 antibody (ab178089, 1:400, Abcam) was used to label CD8^+^ T cell. Anti-CD68 antibody (bs-0649R, 1:400, Bioss), anti-CD163 (ab182422, 1:400, Abcam) and anti-CD206 (60143-1-lg, 1:400, Proteintech) were used to label macrophages. Anti-LPAR5 (bs-15366R, 1:400, Bioss) was used to detect the expression of LPAR5. The immunohistochemical staining images were captured by EVOS FL Auto automatic microscopic imaging system (Life Technologies, US). The immunofluorescence staining images were captured by a high-resolution slide scanning system (Pannoramic MIDI; 3DHISTECH, Hungary). Five high magnification fields were randomly selected for each slide and subsequently averaged for quantitative analysis. We used t-test to test the significance. The study has been approved by the Ethics Committee of Tongji Hospital, Tongji Medical College, Huazhong University of Science and Technology, Wuhan, China (TJ-IRB20211241).

### 2.12 Pan-cancer analysis of LPAR5

To investigate the expression of LPAR5 in other tumors and its impact on prognosis, we further analyzed all kinds of tumors in the TCGA by using the GEPIA2 database (43) (http://gepia2.cancer-pku.cn/#index) and TIMER 2.0 database (44) (http://timer.comp-genomics.org/).

## 3 Results

### 3.1 ESTIMATE analysis

**Figure 2:**
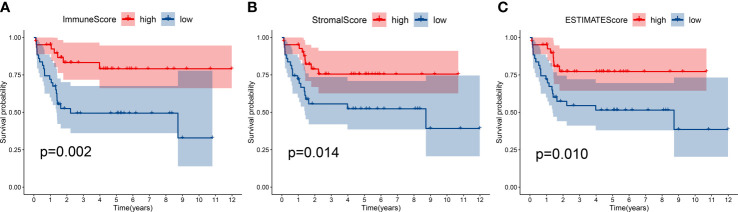
Kaplan-Meier survival analysis. Patients with a higher immune score **(A)**, stromal score **(B)** and ESTIMATE score **(C)** had a better outcome.

Immune score, stromal score and ESTIMATE score were determined by using the ESTIMATE algorithm. Kaplan–Meier (K-M) analysis showed that patients with a higher immune score, stromal score and ESTIMATE score had a better outcome (**Figures 2A-C**), implied immune components in TME might have a positive effect on survival, and might be a suitable indicator for predicting the prognosis of osteosarcoma patients.

### 3.2 Identification of DEGs

**Figure 3:**
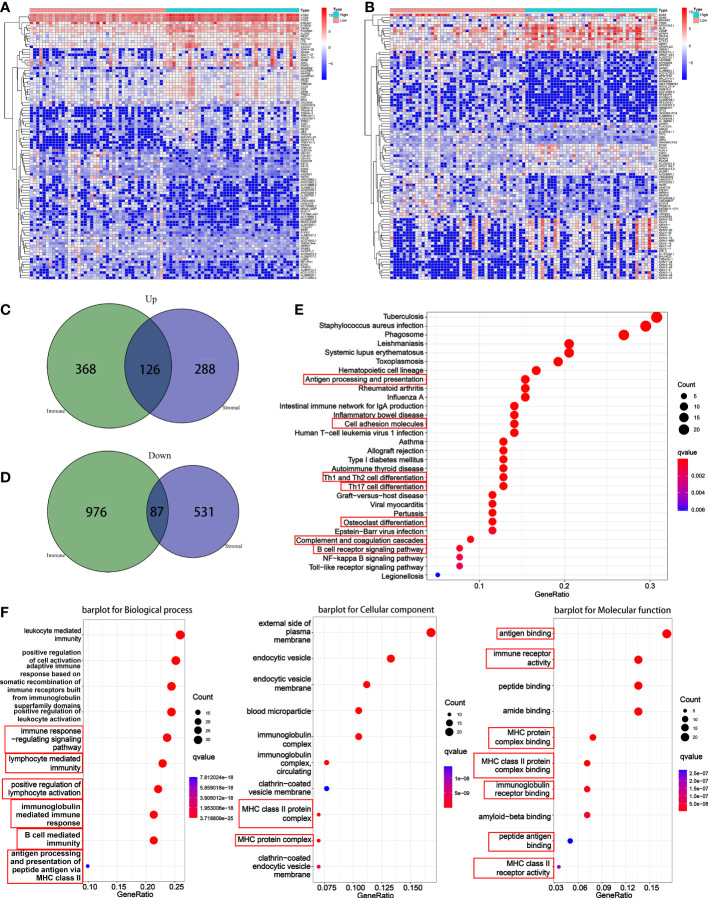
Differential analysis and enrichment analysis based on immune score and stromal score. **(A)** A heatmap for the top 50 up-regulated DEGs and the top 50 down-regulated DEGs by comparison of the high immune score group to the low immune score group. **(B)** A heatmap for the top 50 up-regulated DEGs and the top 50 down-regulated DEGs by comparison of the high stromal score group to the low stromal score group. **(C)** A venn diagram showed common up-regulated DEGs. **(D)** A venn diagram showed common down-regulated DEGs. **(E)** KEGG enrichment analysis based on overlapped DEGs. **(F)** GO enrichment analysis based on overlapped DEGs.

In order to explore the DEGs in TME, immune components and matrix components were analyzed, respectively. When come to immune score, 1557 DEGs (494 genes up-regulated, and 1063 down-regulated) were obtained (**Supplementary Table S1**). When come to stromal score, 1032 DEGs (414 genes up-regulated and 618 down-regulated) were obtained (**Supplementary Table S2**). The heatmap showed the top 50 up-regulated and the top 50 down-regulated DEGs (**Figures 3A, B**). Intersection analysis showed a total of 126 genes were up-regulated both in immune score and stromal score, and 87 genes down-regulated relatively (**Figures 3C, D**).

### 3.3 Functional enrichment analysis

For KEGG pathway, results indicated that the DEGs were enriched into antigen processing and presentation, cell adhesion molecules, Th1 and Th2 cell differentiation, Th17 cell differentiation, osteoclast differentiation, complement and coagulation cascades, and B cell receptor signaling pathway (**Figure 3E**). For GO function, results suggested that the DEGs were enriched into the immune-related terms, such as immune response-regulating signaling pathway, lymphocyte mediated immunity, positive regulation of lymphocyte, MHC class II protein and associated functions, B cell-mediated immunity, antigen binding and processing and so on (**Figure 3F**).

### 3.4 Identification of key genes

**Figure 4:**
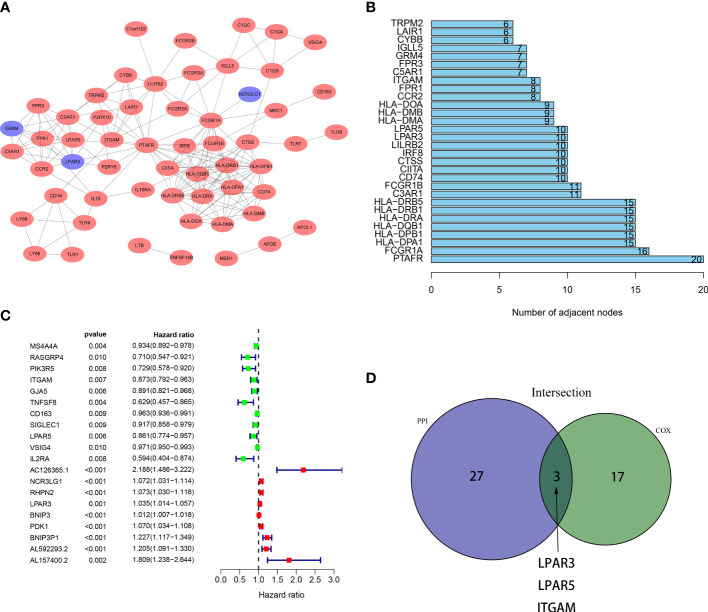
Intersection of results from PPI network and univariate Cox regression. **(A)** A PPI network was constructed based on DEGs with an interaction confidence value >0.90. The red ellipses represent up-regulated genes, and the blue ellipses represent down-regulated genes. **(B)** The top 30 gene nodes in the PPI network. **(C)** Univariate COX regression analysis revealed 20 prognostic genes. **(D)** A venn diagram showed 3 key genes shared by the PPI network and univariate Cox analysis.

Key genes associated with prognosis were obtained through intersection analysis of PPI network and univariate Cox regression. Firstly, we constructed a PPI network, counted the number of nodes owned by each gene (**Figure 4A** **)**, and took the top 30 key genes as key node genes (**Figure 4B** **)**. Then univariate Cox analysis identified 20 prognostic genes (**Figure 4C**). At last, 3 genes, LPAR3, LPAR5, ITGAM, were screened out through intersection analysis (**Figure 4D**). Through literature consulting, we found that among the 3 genes, LPAR5 may be a potential target. Therefore, we focused on LPAR5 in subsequent work.

### 3.5 Survival analysis and clinical correlation analysis

**Figure 5:**
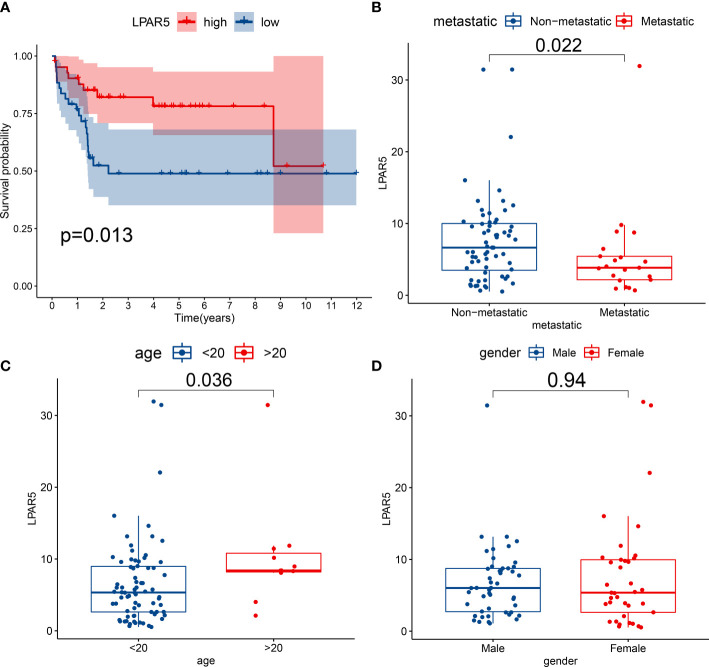
Survival analysis and clinical correlation analysis. **(A)** Survival analysis showed patients with high LPAR5 expression had a better overall survival. **(B)** The correlation between LPAR5 and metastasis. **(C)** The correlation between LPAR5 and age. **(D)** The correlation between LPAR5 and gender.

Patients were divided into the high expression group and the low expression group according to LPAR5 expression. Survival analysis showed patients with high LPAR5 expression had a better overall survival (P<0.05) (**Figure 5A**). Clinical correlation analysis showed that LPAR5 was significantly associated with metastasis and age (P<0.05) (**Figures 5B, C**). But LPAR5 had no relationship with gender (**Figure 5D**).

### 3.6 GSEA analysis

**Figure 6:**
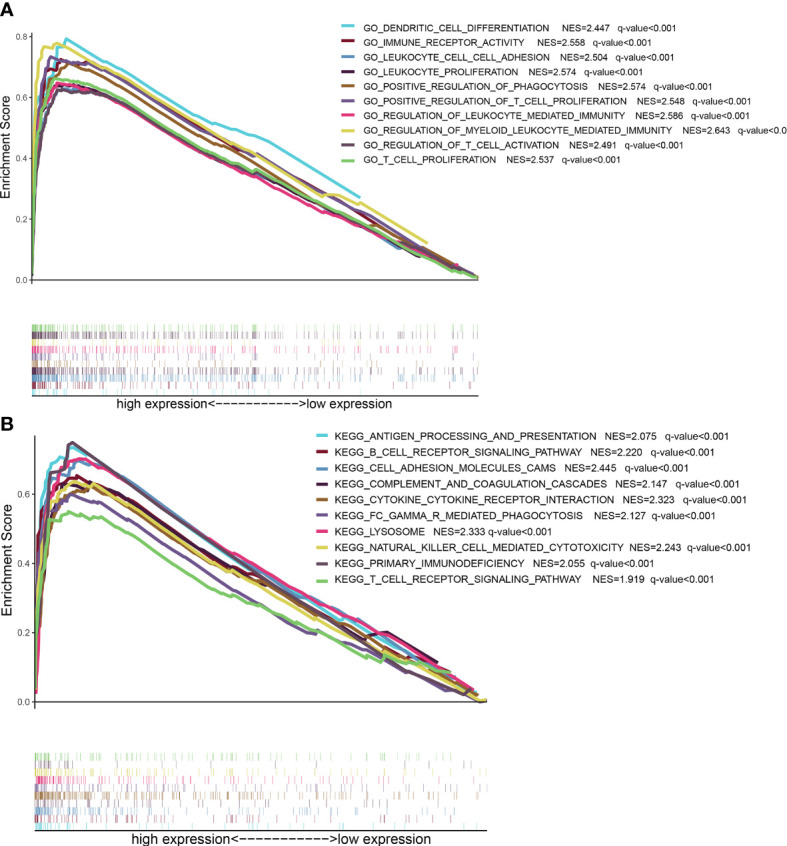
GSEA analysis based on LPAR5 expression. **(A)** The enriched gene sets of high LPAR5 expression group in GO collection. **(B)** The enriched gene sets of high LPAR5 expression group in KEGG collection.

GSEA analysis showed that LPAR5 was related to immune activities, including apoptosis, component, proliferation and activation of T cells and secretion of cytokines. And these functions and pathways were mainly enriched in LPAR5 high-expression group (**Figures 6A, B**).

### 3.7 Immune-infiltration analysis

**Figure 7:**
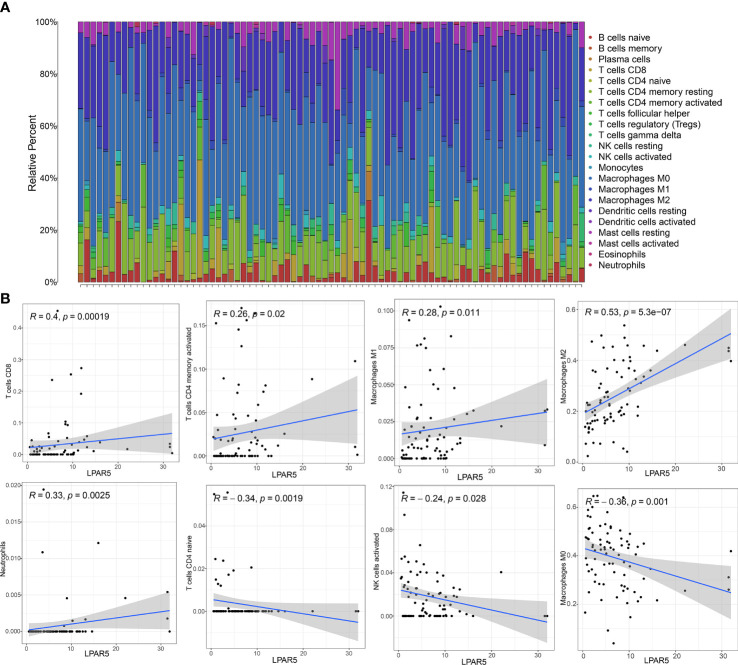
Immune-infiltration analysis by using CIBERSORT algorithm. **(A)** A barplot showing the proportion of TIICs in all osteosarcoma samples. **(B)** A scatter plot showed the correlation of CD8^+^ T cells, CD4^+^ activated memory T cells, M1 macrophages, M2 macrophages, neutrophiles, CD4^+^ naive T cells, activated NK cells and M0 macrophages with the LPAR5 expression.

Immuno-infiltration analysis by using CIBERSORT algorithm showed that macrophages and T cells were the main components of all the samples (**Figure 7A**). Through correlation analysis, we found that the expression of LPAR5 was significantly positively correlated with CD8^+^ T cells (R=0.40, p<0.001), CD4^+^ activated memory T cells (R=0.26, p=0.02), M1 macrophages (R=0.28, p=0.011), M2 macrophages (R=0.53, p<0.001), neutrophils (R=0.33, p=0.0025), and negatively correlated with naive CD4^+^ T cells (R=-0.34, p=0.0019), activated NK cells (R=-0.24, P=0.028) and M0 macrophages (R=-0.36, p=0.001) (**Figure 7B**), which supported the suppose that the expression of LPAR5 was related to the immune response of TME.

### 3.8 Survival analysis of LPAR5-expressing immune cells

**Figure 8:**
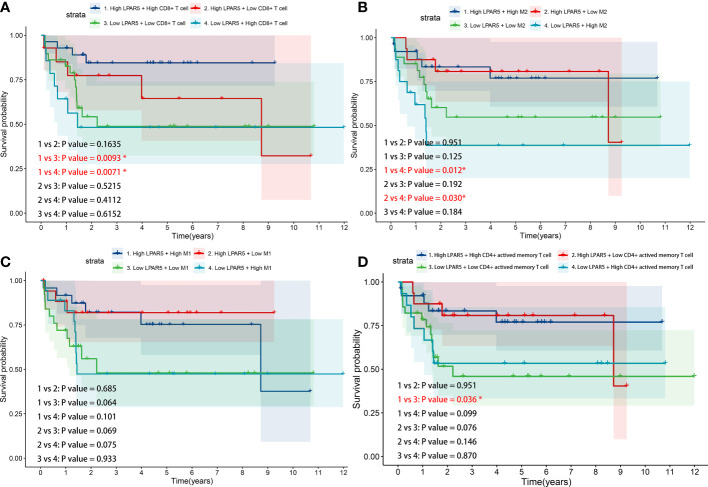
Survival analysis of LPAR5-expressing immune cells. **(A)** CD8^+^ T cell. **(B)** M2 macrophage. **(C)** M1 macrophage. **(D)** CD4^+^ activated memory T cell.

From the results above, it was clear that high LPAR5 expression predicted a better prognosis, and correlation analysis also showed a positive correlation between LPAR5 and CD8^+^ T cells, CD4^+^ activated memory T cells and macrophages; therefore, we further investigated whether immune cells with high LPAR5 expression have an impact on survival. The results showed that patients in the High LPAR5 + High CD8^+^ T cell group exhibited significantly better survival than those in the Low LPAR5 + Low CD8^+^ T cell group and Low LPAR5 + High CD8^+^ T cell group (**Figure 8A**). Patients in the Low LPAR5 + High M2 Macrophage group exhibited significantly worse survival than those in the High LPAR5 + High M2 Macrophage group and High LPAR5 + Low M2 Macrophage group **(** **Figure 8B**). M1 macrophages had a similar pattern, but the p-values were not statistically significant (**Figure 8C**). Patients in the High LPAR5 + High CD4^+^ activated memory T cell group exhibited significantly better survival than those in the Low LPAR5 + Low CD4^+^ activated memory T cell group (**Figure 8D**). These results suggest, in some degree, that LPAR5^+^ immune cells portend a better prognosis.

### 3.9 Analysis and validation in GEO datasets

**Figure 9:**
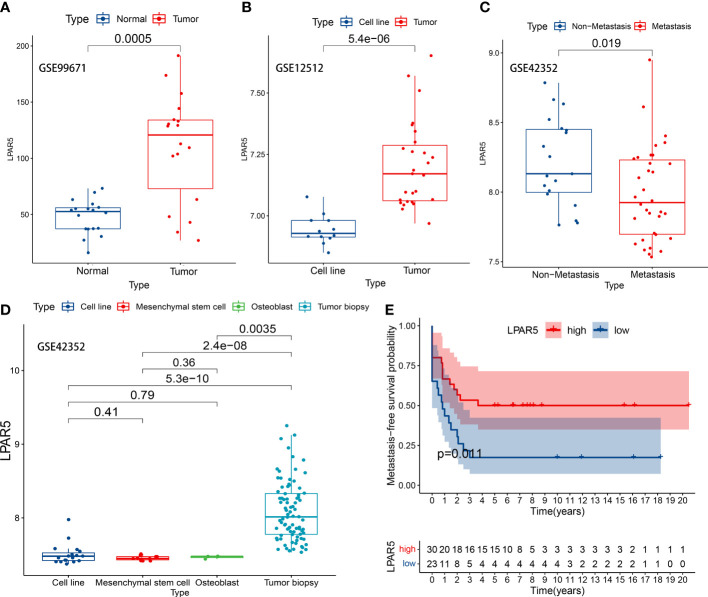
LPAR5 expression of osteosarcoma in GEO datasets. **(A)** LPAR5 expression between tumor tissues and adjacent normal tissues in GSE99671 dataset. **(B)** LPAR5 expression between tumor tissue and osteosarcoma cell line in GSE12512 dataset. **(C)** Validation of the difference of LPAR5 expression among osteosarcoma biopsy, osteosarcoma cell line, mesenchymal stem cell and osteoblast by GSE42352 dataset. **(D)** Validation of the difference of LPAR5 expression between metastasis patients and non-metastasis patients by GSE42352 dataset. **(E)** Validation of the prognostic value of LPAR5 in osteosarcoma patients by GSE21257.

In GSE99671 dataset, the expression of LPAR5 was higher in the tumor tissues (P<0.001) (**Figure 9A**). In GSE12512 dataset, the expression of LPAR5 was higher in tumor tissue than in osteosarcoma cell line (P<0.001) (**Figure 9B**). In GSE42352 dataset, it was also found that LPAR5 had higher expression in non-metastasis patients than metastasis patients (P=0.019) (**Figure 9C**), and the expression of LPAR5 was much higher in tumor biopsy than cell line, mesenchymal stem cell and osteoblast (P<0.001) (**Figure 9D**), which was consistent with the results we found above. These results also suggested that LPAR5 was predominantly expressed by immune components or stromal components of the TME rather than tumor cells.

To verify the effect of LPAR5 on the prognosis of patients with osteosarcoma, dataset GSE21257 contained prognostic information was used for analysis. We analyzed the impact of LPAR5 on the prognosis of osteosarcoma patients by metastasis-free survival as defined by the authors of the dataset (37). Patients were divided into LPAR5 high expression group and low expression group. The optimal cut-off value was 8.40, which was determined by the X-tile software (Version 3.6.1) (45). Detailed information was in **Supplementary Table S3**. The results found that patients with higher expression of LPAR5 had better metastasis-free survival (**Figure 9E**) (P<0.05).

**Figure 10:**
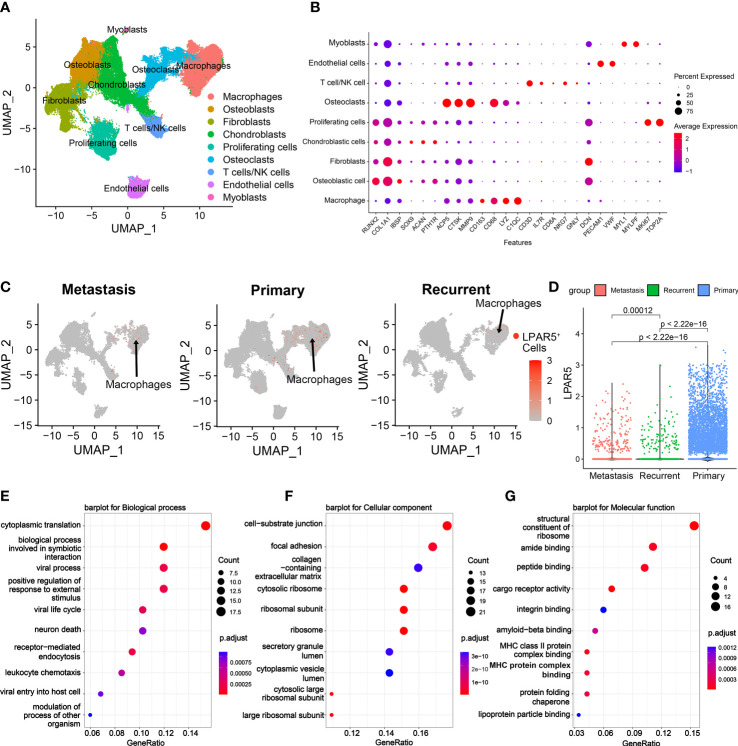
Single cell RNAseq analysis. **(A)** UMAP plot showing the cell clusters in the TME. **(B)** Expression of marker genes of cells in the TME. **(C)** UMAP plot showing cell clusters, which expressed LPAR5, in the metastasis patients, the primary patients and the recurrent patients, respectively. The gray dots indicate LPAR5-negative cells. The red dots represent the LPAR5-positive cells, and the darker the color, the higher the expression of LPAR5. **(D)** Violin plot showed that LPAR5 expression was higher in primary patients than metastasis patients and recurrence patients. **(E)** A barplot for biological process. **(F)** A barplot for cellular component. **(G)** A barplot for molecular function.

To investigate the site of LPAR5 expression in osteosarcoma tissues, a single-cell dataset (GSE152048) was used for further analysis. Uniform Manifold Approximation and Projection (UMAP) analysis was performed for dimension reduction, and the cells were overlapped into 9 cell clusters, namely macrophages, osteoblasts, fibroblasts, chondroblasts, proliferating cells, osteoclasts, T cells/NK cells, endothelial cells, myocytes (**Figure 10A**), according to the cell markers (**Figure 10B**). LPAR5 was predominantly expressed in the macrophage cluster, and a very small amount of LPAR5 was also expressed in the osteoclast cluster (a kind of macrophages) (46, 47) and T/NK cell cluster (**Figure 10C**). What’s more, LPAR5 expression was much higher in primary osteosarcoma patients than metastasis patients and recurrent patients (**Figure 10D**, **Table 1**). Then macrophages were divided into LPAR5^+^ macrophages and LPAR5^-^ macrophages, and the 124 DEGs (including LPAR5) was screened out with P<0.05. Functional enrichment analysis was performed based on these DEGs. The results showed that these DEGs were enriched in the biological processes associated with phagocytosis and antigen presentation (**Figures 10E-G**).

**Table 1:** LPAR5^+^ macrophages among primary, metastasis and recurrent osteosarcoma patients.

	Positive	Negative	Total	Positive Rate
Primary	1682	17206	18888	8.91%
Metastasis	92	1526	1618	5.69%
Recurrence	79	1539	1618	4.88%
Total	1853	20271	22124	8.38%

×^2^ = 47.878, Degree of Freedom = 2, P <0.0001.

### 3.10 Immunohistochemical and immunofluorescence staining

The slides of 12 patients were analyzed, and the basic information of the patients is shown in the **Supplementary Table S4**. Seven of the twelve patients had progression disease (PD) and the other five patients had no progression disease (NPD). PD was defined as enlargement of the primary tumor, and/or the development of new lesions and/or metastasis in the course of primary treatment.

**Figure 11:**
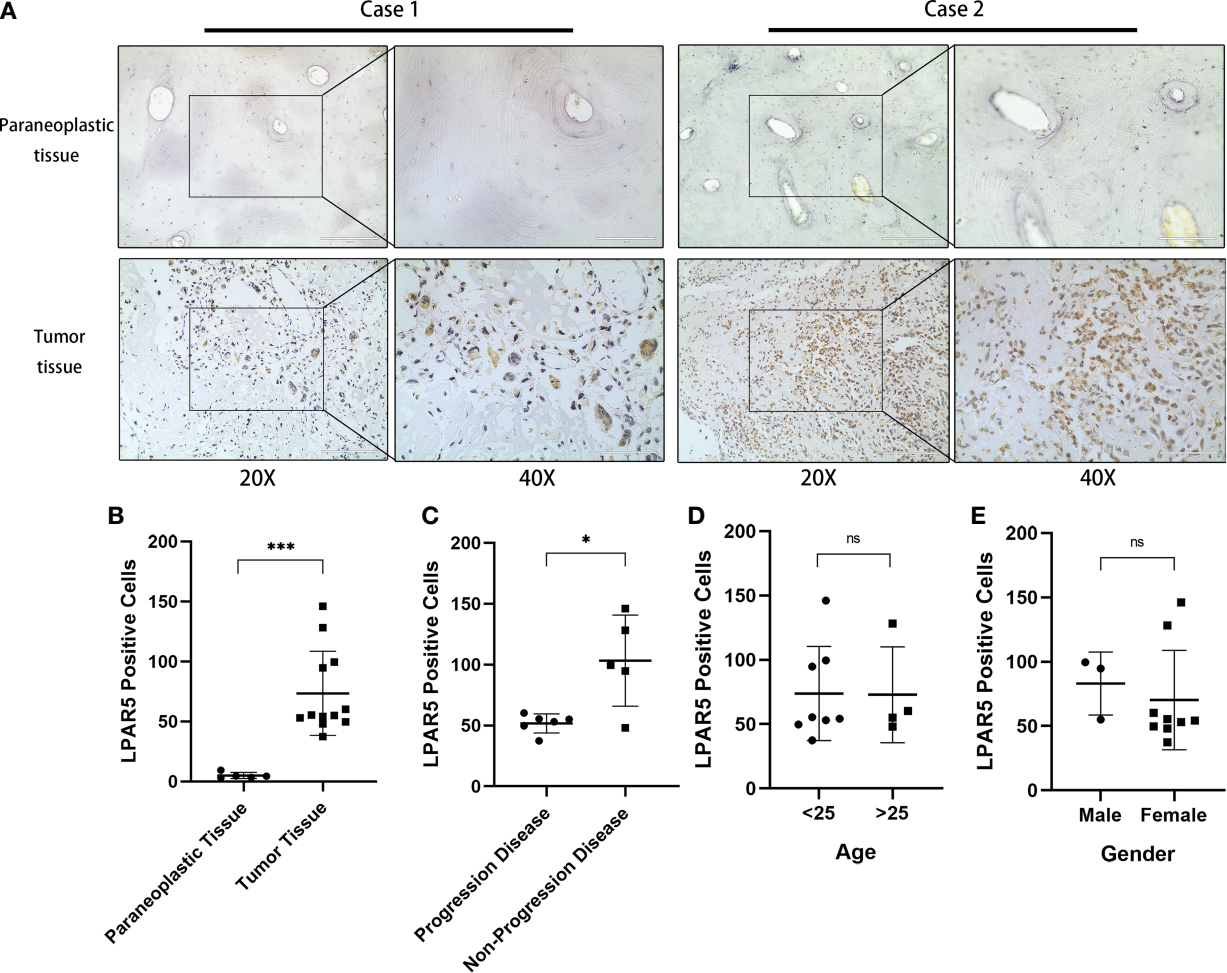
Immunohistochemical staining. **(A)** Immunohistochemical staining of LPAR5 in osteosarcoma sample and normal sample. Scale bar (20× = 200μm, 40× = 100μm). **(B)** The difference of LPAR5 expression in tumor and paraneoplastic tissues. **(C)** The difference of LPAR5 expression in progression disease patient and no progression disease patients. **(D)** LPAR5 expression in patients under 25 years of age and patients over 25 years of age. **(E)** LPAR5 expression between male and female. *: P<0.05; ***: P<0.001; ns, not significant.

By immunohistochemical staining, it could be found that LPAR5 was highly expressed in tumor tissues than paraneoplastic tissues (P<0.001), and LPAR5 was expressed both in multi-nucleated cells and mono-nucleated cells. (**Figures 11A, B**). By quantitative analysis, we found that the number of LPAR5 positive cells was significantly higher in NPD patients than in PD patients (P<0.05) (**Figure 11C**), which was consistent with the results of our bioinformatics analysis. However, there was no difference in age (P>0.05) (**Figure 11D**) and gender (P>0.05) (**Figure 11E**). This may be due to the small sample size.

**Figure 12:**
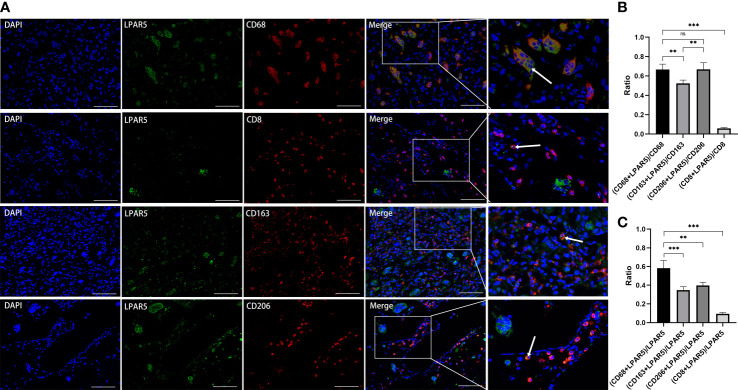
Immunofluorescence staining. **(A)** Immunofluorescence staining of LPAR5, CD68, CD163, CD206 and CD8. **(B)** The proportion of LPAR5 positive expression in CD68+ macrophages, CD163^+^ macrophages, CD206^+^ macrophages and CD8^+^ T cells. **(C)** Comparison of CD68^+^ macrophages, CD163^+^ macrophages, CD206^+^ macrophages and CD8^+^ T cells in LPAR5-positive cells. Co-localization was highlighted with white arrows. Scale bars: 100μm. **: P<0.01; ***: P<0.001; ns, not significant.

Subsequent results of immunofluorescence staining showed that LPAR5 was mainly expressed on macrophages, and a very small amount on CD8^+^ T cells (**Figure 12A**), which was consistent with our previous bioinformatics analysis. Among the labelled macrophages, CD68^+^ macrophages were almost all multi-nucleated cells, while CD163^+^ and CD206^+^ macrophages were almost all mono-nucleated cells. We calculated the percentage of LPAR5 positivity in each cell and compared the ratio of CD68^+^ macrophages, CD163^+^ macrophages, CD206^+^ macrophages and CD8^+^ T cells in LPAR5-positive cells. We found that LPAR5 was expressed on CD68^+^/CD206^+^ cells (M2 macrophages), CD68^+^/CD163^+^ cells (M2 macrophages) and CD68^+^/CD206^-^/CD163^-^ cells (M1 macrophages) (**Figures 12B, C**), and accounted for the majority of LPAR5-positive cells, indicating that LPAR5 was not restricted to M1 or M2 macrophages. This also suggested that LPAR5 is specifically expressed on macrophages.

### 3.11 Pan-cancer analysis of LPAR5

**Figure 13:**
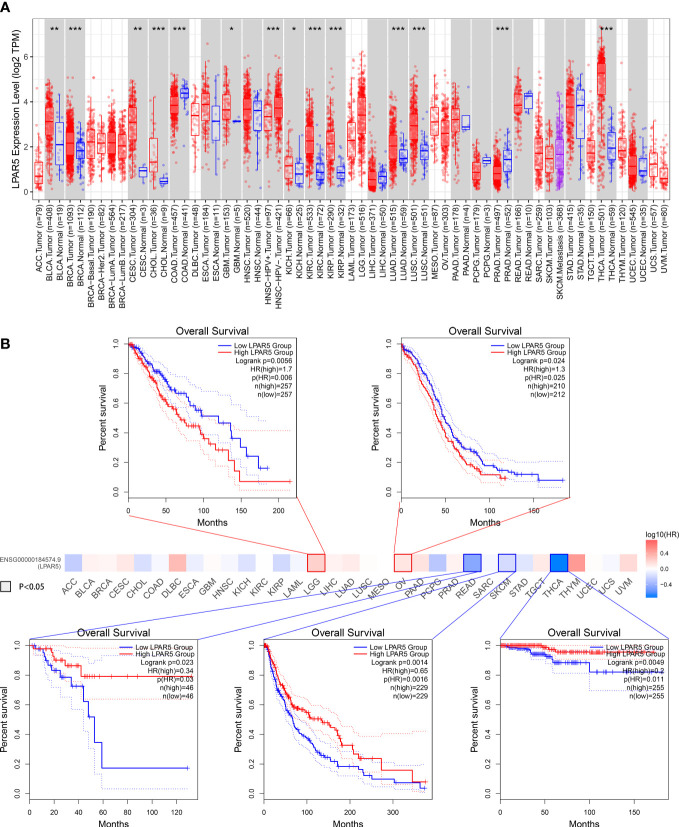
Pan-cancers analysis. **(A)** LPAR5 expression in pan-cancers. **(B)** Survival analysis in pan-cancers. *: P<0.05; **: P<0.01; ***: P<0.001; ns, not significant.

We further analyzed all tumors in the TCGA using the GEPIA2 and TIMER 2.0 database. The result showed that LPAR5 was differentially expressed in many tumors. In Bladder Urothelial Carcinoma (BLCA), Breast invasive carcinoma (BRCA), Cervical squamous cell carcinoma and endocervical adenocarcinoma (CESC), Cholangiocarcinoma (CHOL), Glioblastoma multiforme (GBM), Kidney Chromophobe (KICH), Kidney renal clear cell carcinoma (KIRC), Kidney renal papillary cell carcinoma (KIRP), Lung adenocarcinoma (LUAD), Lung squamous cell carcinoma (LUSC), Thyroid carcinoma (THCA), LPAR5 was highly expressed in tumor tissue. In Colon adenocarcinoma (COAD), Prostate adenocarcinoma (PRAD), LPAR5 was highly expressed in normal tissue (**Figure 13A**). Subsequent survival analysis showed that in Brain Lower Grade Glioma (LGG) and ovarian serous cystadenocarcinoma (OV), high LPAR5 expression predicted a worse prognosis, whereas in Rectum Adenocarcinoma (READ), Skin Cutaneous Melanoma (SKCM) and Thyroid carcinoma (THCA), high LPAR5 expression predicted a better prognosis (**Figure 13B**).

## 4 Discussion

The development of a tumor includes *in situ* growth, invasion, extravasation and metastasis (48). During these processes, tumor cells would interact with components of their microenvironment, such as immune cells, stromal cells and inflammatory cells (49). Different proportions of lymphocytes, macrophages, and inflammatory cells are often found in the pathological slides of tumors. Inflammatory cells and tumor-associated macrophages were thought to produce factors that maintain chronic inflammation and promote tumor growth (50, 51). Lymphocytes might play an essential role in inhibiting tumor growth and metastasis (52–54). Immune cells and inflammatory cells in TME may be potential therapeutic targets (55–57). As a result, it is essential to explore the potential indicators and therapeutic targets of TME, changing the proportion of TIICs, and inhibiting tumor development.

In this study, we attempted to identify key TME-related genes that could help predict survival and may be potential therapeutic targets. Firstly, the immune score, stromal score and ESTIMATE score were calculated by using the ESTIMATE algorithm, and the results showed that immune score, stromal score and ESTIMATE score were all highly correlated with overall survival, indicating that the proportion of immune components and stromal components in TME was an important prognostic factor. Patients were grouped according to the immune score and stromal score. Then the DEGs were screened out, and we found that DEGs were mainly mapped into the biological activities and pathways related to immune response, suggesting that the immune response is essential for TME modeling. The DEGs were used to construct PPI networks to find core nodes. Meanwhile, prognostic genes were selected by Cox regression analysis. Combining the results, we obtained three key genes. Through subsequent research and analysis, we found that LPAR5 was a promising indicator for TME remodeling with a prognostic value for osteosarcoma.

LPAR5 is a receptor of lysophosphatidic acid (LPA). LPA is a bioactive phospholipid with mitogenic and growth factor-like activities (58). Overwhelming evidence indicates that LPA signaling serves vital roles in a wide range of physiological effects involving cell proliferation, cancer progression, metastasis and drug resistance, which were modulated by interacting with six G protein-coupled receptors, LPAR1–6 (59). These LPARs could be divided into endothelial cell differentiation gene (EDG) families and non-EDG families according to their molecular structure (60). However, different receptors regulate distinct functions; the role of LPARs in cancer remains controversial (61). LPAR5 belongs to the non-EDG family and might act as a tumor suppressor. LPAR5 was found to act as an anti-migratory receptor *via* cAMP-PKA pathway in B16 melanoma cells (62). Araki et al. suggested that LPA signaling *via* LPAR5 inhibited cell motility activity of rat sarcoma cells (63). Ishii et al. found that LPAR5 knockdown stimulated the malignant properties, such as cell motility, invasion, tumorigenicity and angiogenesis in PANC-1 cells (64). LPAR5 was also found down-regulated in primary nasopharyngeal carcinoma, and the downregulation of LPAR5 promoted the LPA-induced migration of nasopharyngeal carcinoma cell lines (65). In the present study, we found that LPAR5 expression was positively correlated with overall survival and negatively correlated with metastasis, indicating LPAR5 might be protective factors for osteosarcoma patients. In addition, osteosarcoma is commonly found in children and adolescents. Our study found statistically significant differences in LPAR5 expression between patients over and under 20 years of age in TCGA set, which may provide some ideas as to why LPAR5 express predominantly in children and adolescents. However, we did not observed the same result in the correlation analysis for clinico-pathological data from immunochemical staining. This may be due to the small sample size. Similar results were also found in READ, SKCM and THCA through pan-cancer analysis. This is consistent with these studies above. However, pan-cancer analysis also found that high LPAR5 expression predicted a worse prognosis in LGG, OV and PAAD. Some studies have also reported the pro-tumor effect of LPAR5. Zheng et al. found LPAR5 was up-regulated in breast carcinoma samples with higher rates of metastasis (66). Wu et al. showed that the downregulation of LPAR5 expression could inhibit the physiological process of papillary thyroid cancer *via* PI3K/AKT pathway and epithelial-mesenchymal transition (EMT) process (67). These results suggest that the role of LPAR5 may be different in different tumors. However, LPAR5 was rarely studied before in osteosarcoma. There was only one study reported that the motile activity of osteosarcoma cells was inhibited by LPAR5 knockdown (68), which was the opposite of our results. But the effect of LPAR5 in the study was reported to mediate by endothelial cells and anti-tumor drugs. The effect of LPAR5 on the TME was not considered. We hypothesized that LPAR5 might affect the composition of immune cells and stromal cells in TME, thus affecting tumor development. By the comparison between the tumor tissues and paired normal bone tissues, we found that the expression of LPAR5 was higher in the tumor tissues, which was also validated by immunochemical staining. In addition, the expression of LPAR5 was higher in tumor tissue than in osteosarcoma cell line. These results supported our speculation that LPAR5 may not be expressed by osteosarcoma cells but by immune components or stromal components in TME. We subsequently confirmed that LPAR5 was predominantly expressed in macrophages by using single cell RNAseq data and immunofluorescence staining. Therefore, we focused on its possible role in the TME in the following investigation.

LPARs were reported to express in various immune cells, including lymphocytes (69, 70) and dendritic cells (71, 72). In addition, LPAR5 was positively correlated with trafficking, survival and communication of immune cell subpopulations (73). The results of GSEA showed that genes in LPAR5 high expression group were mainly enriched in immune-related biological processes and pathways. Thus, LPA signaling through LPAR5 can act as an important regulatory factor in the immune system. LPAR5 was reported to express in T cells and macrophages (74), which was consistent with our results. It is well known that CD8^+^ T cells play a primary role in anti-tumor immunity (75). CD4^+^ T cells have both tumor-inhibiting activity and tumor-promoting activity, depending on the cell subpopulation type and immune microenvironment (76). Many studies have suggested that M2 polarized tumor-associated-macrophages (TAM) are associated with tumor growth, invasion, and metastasis (77–79). In addition, TAM can also suppress the immune response by secreting inhibitory factors (80). In our study, it was found that LPAR5 expression was positively correlated with CD8^+^ T cells, CD4^+^ activated memory T cells, M1 macrophages and M2 macrophages, and patients with higher LPAR5 expression had a better outcome. However, in single cell analysis and immunofluorescence staining, it was found that LPAR5 was predominantly expressed in macrophages and a very small amount expression in T/NK cells, which implied the infiltration of CD8^+^ T cells was not directly related to high expression of LPAR5. And this could also explain why the expression of LPAR5 level is higher in tumor tissues than in normal tissues. The result of GSEA showed that LPAR5 in macrophages mainly correlated to phagocytosis and antigen presentation associated functions. As a result, we speculate that LPAR5 might enhance phagocytosis and antigen presentation of macrophages, recruiting more CD8^+^ T cells and CD4^+^ activated memory T cells to infiltrate the tumor microenvironment and inhibiting tumor cell growth. It was reported that M1 macrophages promoted inflammatory responses, were capable of antigen presentation and the activation of T cells, and therefore have anti-tumor and anti-metastatic effects (81, 82). Manuel Weber et al. compared the difference in macrophage infiltration between craniofacial osteosarcoma (COS) and extracranial osteosarcoma (EOS) and found that M1 macrophages were more infiltrated in COS, which could explain the low probability of metastasis in COS (83). M2 macrophages were reported to have immunomodulatory effects and were associated with wound healing, immunosuppression, tumor progression and metastasis (84–86). However, Anne Gomez-Brouchet et al. found that the presence of CD163-positive M2-polarized macrophages was essential to inhibit osteosarcoma progression (87). This implies that M2-polarized macrophages may have different pro- or anti-tumor effects influenced by other factors. Zhang et al. stated that the polarization level of M0 to M1 or M2 macrophages may be an important factor (88). It has also been reported that the balance between M1 and M2 macrophage might affect the balance of PD-1/PDL-1 system, which is known to be an important immune regulatory system (89). Taking together, it was difficult to clarify the role of M1 and M2 macrophages in osteosarcoma. In our study, we identified LPAR5^+^ macrophages that were present in the tumor tissue, and higher expression predicted a better prognosis, which would provide a new insight into prognosis assessment and immunotherapy of osteosarcoma. In addition, the clinical detection of LPAR5^+^ macrophages in surgically resected tumor samples from patients may help in the prediction of tumor metastasis and survival of patients. However, the specific mechanisms of action of LPAR5^+^ macrophages in TME were not investigated in depth. More in-depth basic research is needed at a later stage.

## Conclusion

In this study, we analyzed the TME of osteosarcoma using ESTIMATE and CIBERSORT algorithms and screened out a TME-related gene, LPAR5, which is a promising indicator for TME remodeling in osteosarcoma. Particularly, LPAR5^+^ macrophages might have great potential to be a prognostic factor and therapeutic target for osteosarcoma.

## Data availability statement

The datasets analyzed for this study can be found in the UCSC Xena database (http://xena.ucsc.edu/) and GEO dataset (https://www.ncbi.nlm.nih.gov/geo/) under the accession numbers GSE99671, GSE12512, GSE42352 and GSE152048.

## Ethics statement

The studies involving human participants were reviewed and approved by Ethics Committee of Tongji Hospital, Tongji Medical College, Huazhong University of Science and Technology, Wuhan, China. Written informed consent from the participants’ legal guardian/next of kin was not required to participate in this study in accordance with the national legislation and the institutional requirements.

## Author contributions

HY conceived and supervised the study. YH, HZ and YQ performed the bioinformatics analysis. YW, WP, RZ, SC and XH helped disposal data. YH and HZ wrote the manuscript. HY and XH made manuscript revisions. All authors contributed to the article and approved the submitted version.

## Supplementary material

Supplementary Table 1 DEGs between high immune score group and low immune score group.

Supplementary Table 2 DEGs between high stromal score group and low stromal score group.

Supplementary Table 3 LPAR5 expression status of patients in GSE21257.

Supplementary Table 4 Basic information of patients whose pathological slides underwent immunochemical staining and immunofluorescence staining.
